# Late gestation fetal hypothyroidism alters cell cycle regulation across multiple organ systems

**DOI:** 10.1186/s12917-024-04102-y

**Published:** 2024-06-20

**Authors:** Alyssa A. Smith, Alexa Vesey, Caden Helfrich, J. Alex Pasternak

**Affiliations:** Department of Animal Sciences, Purdue University, West Lafayette, IN 47906 USA

**Keywords:** Fetal, Hypothyroidism, Cell cycle, Late gestation, Methimazole, Cyclin-dependent kinase

## Abstract

**Background:**

Hypothyroidism is a common endocrine disruption observed *in utero* that adversely affects fetal growth and maturation leading to long-term impacts on health; however, the exact molecular mechanisms by which these deleterious effects occur are unknown. We hypothesize that fetal hypothyroidism during late gestation will disrupt cell cycle regulation in a tissue-specific manner. To evaluate this, eight pregnant gilts were dosed with either methimazole or an equivalent negative control during days 85–106 out of 114 days of gestation (*n* = 4/group). Following treatment, the gilts were humanely euthanized, and tissue samples of fetal heart, ileum, kidney, lung, liver, muscle, spleen, and thymus taken from two male and two female fetuses (*n* = 32) from each gilt.

**Results:**

The relative expression of three cell cycle promoters (CDK1, CDK2, and CDK4), and one cell cycle inhibitor (CDKN1A) was compared in each tissue to determine the effect of hypothyroidism on the developing fetus. All of the eight tissues examined experienced at least one significant up- or downregulation in the expression of the aforementioned genes as a result of treatment with methimazole. Substantial changes were observed in the liver and muscle, with the latter experiencing significant downregulations of CDK1, CDK2, and CDK4 as a result of treatment. In addition, all tissues were examined for changes in protein content, which further elucidated the impact of hypothyroidism on the fetal liver by the observation of a marked increase in protein content in the methimazole-treated group. Finally, the heart and liver were histologically examined for evidence of cellular hyperplasia and hypertrophy by measuring average nuclei density and size in each tissue, with the results showing a significant decrease in average nuclei size in the liver of hypothyroid fetuses.

**Conclusions:**

Collectively, these findings indicate the occurrence of organ-specific disruptions in cell cycle progression as a result of *in utero* hypothyroidism, which may explain the long term and widespread effects of hypothyroidism on fetal development.

## Introduction

Thyroid hormones, namely triiodothyronine (T3) and thyroxine (T4), are vital in the regulation of cellular metabolic activities, and thus serve as principal regulators of various physiological systems and processes essential to fetal development. In swine, the fetal thyroid gland becomes active early in gestation to produce substantial amounts of thyroid hormones [1], a required process given the placental-enzymatic barrier that prevents efficient transplacental transmission of maternal thyroid hormones [2]. When the production of thyroid hormones is disrupted, this results in a multitude of detrimental effects in the developing fetus, and while this endocrine disruption is commonly observed during human pregnancy [3], the exact molecular alterations that occur as a result of congenital hypothyroidism are still unclear.

The impact of hypothyroidism *in utero* has been extensively studied in rodents using antithyroid medications such as methimazole (MMI) and propylthiouracil, which are drugs that inhibit thyroid hormone production by interfering with the activity of thyroid peroxidase [4]. Such studies have clearly demonstrated deleterious effects of hypothyroidism including altered neural development [5] and modulations in other fetal endocrine systems [6, 7]. Studies in thyroidectomized fetal sheep have additionally shown that hypothyroidism alters muscle composition and functionality [8], as well as bone development and ossification [9]. These effects are consistent with the long-term implications of fetal hypothyroidism in humans, with observed clinical symptoms such as mental retardation [10], delayed bone development [11], and poor growth [12]. As swine are a commonly used biomedical model [13], the current study utilizes a porcine model of congenital hypothyroidism in which MMI, which we have previously shown to cross the porcine placenta and induce severe fetal hypothyroidism [14], is used to examine the molecular impact of late gestation hypothyroidism on the developing fetus.

Previously, we have shown that *in utero* physiological stressors such as infection with porcine reproductive and respiratory syndrome virus (PRRSV) induces fetal hypothyroidism [15], which is coincident with the dysregulation of cell cycle across multiple tissues [16]. Given the immunological response following fetal PRRSV infection, the observed disruptions in cell cycle progression cannot be directly associated with the concurrent hypothyroidism. Thus, further investigation within a non-pathogenic model is required. The cell cycle is a multi-step process that consists of various stages including gap phase 0 (G0), gap phase 1 (G1), the DNA synthesis stage (S), gap phase 2 (G2), and mitosis (M). G0 is a non-essential rest period where the cell is neither undergoing nor actively preparing for mitosis. This is succeeded by G1, which is a necessary period of growth in preparation for the S stage of the cell cycle, which is when the cell synthesizes the needed DNA for mitotic division. A final period of growth termed G2 then occurs before the M phase, where the cell will complete mitosis and divide to produce equivalent daughter cells. Each step of this process is regulated by the activity of cyclin-dependent kinases (CDKs), which are enzymes that function by phosphorylating their target proteins to modulate their activity [17]. Well-known and understood CDK cell cycle promoters include CDK1, which promotes the transition from G2 to M, CDK2, which promotes the transition from G1 to S, and CDK4, which promotes the transition from G0 to G1 or S [18]. In addition, a primary inhibitor of all of these CDKs is CDKN1A, classically referred to as P21, which has been extensively studied for its relation to cancer and apoptosis [19].

During late gestation, disruptions in the cell cycle may be particularly detrimental, as this is a period of exponential growth [20] in which the fetus must undergo successful terminal maturation to allow for a viable transition to extrauterine life. Thus, the present study sought to characterize the alterations in cell cycle that occur as a result of late gestation fetal hypothyroidism by examining a wide array of tissues including the fetal heart (HRT), ileum (ILE), kidney (KID), lung (LNG), liver (LVR), longissimus dorsi muscle (MUS), spleen (SPLN), and cervical thymus (THY). A targeted gene expression approach was first used in all eight tissues to measure relative changes in the expression of CDKs as a result of treatment with MMI. All eight tissues were additionally examined for changes in protein content to further elucidate the molecular effects of congenital hypothyroidism on the developing fetus. Finally, the LVR and HRT were histologically evaluated for evidence of cellular hyperplasia and hypertrophy as a result of MMI treatment. Based on prior observation of alterations in the expression of CDKs as a result of fetal PRRSV infection and the associated hypothyroidism [16], we hypothesized that goitrogen-induced fetal hypothyroidism would have widespread effects on the gene expression of key cell cycle regulators, which may explain some of the detrimental effects of hypothyroidism on the developing fetus.

## Materials and methods

### Animal model

The non-pathogenic model of porcine fetal hypothyroidism used in the present study was described in detail in Ison et al. 2023 [14]. In short, eight terminal cross gilts (Landrace x Large White dams and Duroc sires) were selected from the Animal Sciences Research and Education Center (ASREC) at Purdue University. Following confirmation of first estrus, gilts were synchronized with 17.6 mg of Altrenogest per day for 17 days (Merck Animal Health, Kenilworth, NJ, USA) and bred via artificial insemination on the first standing estrus post withdrawal. All animals were confirmed pregnant by transcutaneous ultrasound on gestation day 30 and randomly assigned into either a control (CON) or MMI treatment (*n* = 4/group). Starting on gestation day 85, gilts were provided daily with 200 g of supplemental feed containing 5 mg/kg of MMI (Sigma-Aldrich, St. Louis, MO, USA) or the equivalent sham carrier (50% corn syrup in water). After 21 days of treatment (gestation day 106) the gilts were humanely euthanized via captive bolt and exsanguination in keeping with the American Veterinary Medical Association (AVMA) guidelines for euthanasia. The gravid uterus was carefully extracted, linearized, and the relative positions, sex, and viability of all fetuses (*n* = 120) were recorded using previously established methods [21]. Fetal serum was collected from the axillary artery for assessment of circulating T3 and T4, and the thyroid extracted and fixed in formalin for histological examination, the combination of which was used to verify fetal hypothyroidism within the MMI-treated litters, as shown in Ison et al. 2023 [14]. Samples of fetal HRT, ILE, KID, LNG, LVR, MUS, SPLN, and THY were collected from the most centrally located viable male and female fetuses in each uterine horn, snap frozen in liquid nitrogen, and then stored at -80 °C (*n* = 32). Additional samples of the left ventricle and a central section of the left lateral LVR lobe were fixed in 10% neutral buffered formalin for 48 h before being processed and paraffin embedded by the Purdue Histology Research Laboratory. In addition to the data discussed here, phenotypic parameters and organ weights for all fetuses are discussed in detail in Ison et al. 2023 [14]. All animal procedures were carried out in compliance with Purdue University’s animal care policies and approved by the Institutional Animal Care and Use Committee (IACUC Protocol #2103002122).

### RNA extraction and qPCR

For gene expression analysis, frozen tissue samples from select fetuses were manually ground to a fine powder under liquid nitrogen in a pre-cooled mortar and pestle. Subsequent RNA extraction was then performed using Trizol reagent (Thermo Fisher Scientific, Waltham, MA) and a double precipitation method. After extraction, a 20 µL aliquot of each sample was DNase treated to remove endogenous DNA using a modified protocol of the Turbo DNA-free kit (Thermo Fisher Scientific), with the addition of 0.5 µL of RNaseOUT (Thermo Fisher Scientific) in each reaction. RNA was then quantified using a Nanodrop ND1000 spectrophotometer (Thermo Fisher Scientific) and assessed for integrity using a 1.2% (wt/vol) denaturing agarose gel [22]. Finally, RNA samples were reverse transcribed to produce cDNA using the High-Capacity cDNA Reverse Transcription kit (Thermo Fisher Scientific), with 2 µg of total RNA used per reaction. The resultant cDNA libraries were diluted to a final concentration of 10 ng/µL and stored at -20 °C. Specific primers for reference genes and genes of interest (GOI) were designed to span exon-exon junctions (Table 1), as identified by the BLAST-Like Alignment Tool (BLAT) relative to the Sscrofa11.1 genome assembly. Based on current RefSeq mRNA sequences, these primers were determined to pick up all known transcript variants of each gene and be specific to each gene target, as confirmed by the Primer-Basic Local Alignment Search Tool (Primer-BLAST). Resultant primers were validated using a 5-point serial dilution, with a strict efficiency requirement of 95–105%, and verification of a single amplicon product evident by the production of a single melt curve peak. Once validated, the relative expression of each GOI across tissue was assessed via qPCR on a CFX Connect qPCR system (Bio-Rad, Hercules, CA), with the use of SsoAdvanced Universal SYBR Green Supermix (Bio-Rad) and 20 µg of cDNA per reaction, with each sample assayed in duplicate. Resultant Ct values were normalized using the geometric mean of the 2–3 most stable reference genes in each tissue, and results presented as a fold change for each sample relative to the control group of the tissue using the 2^−ΔΔCT^ method, with results displayed graphically on a log10 scale to clearly show both up and down regulations.

**Table 1:** Porcine specific primer sequences used for qPCR

	Gene ID	Symbol	Forward Primer	Reverse Primer	Amplicon Length	Annealing Temp	Target sequence/ reference
**Reference**	414396	ACTB	5`-CCAGCACGATGAAGATCAAG-3`	5`-AGTCCGCCTAGAAGCATTTG-3`	171	61	[14]
	396823	GAPDH	5`-CCTGGAGAAACCTGCAAAAT-3`	5`-TTGACGAAGTGGTCGTTGAG-3`	183	60	NM_001206359.1
	396581	HMBS	5`-AGGATGGGCAACTCTACCTG − 3`	5`-GATGGTGGCCTGCATAGTCT-3`	83	61	[62]
	397637	PPIA	5`-CACTGCCAAGACTGAGTGGT-3`	5`-TGTCCACAGTCAGCAATGGT-3`	144	61	[21]
	396989	RPL19	5`-AACTCCCGTCAGCAGATCC-3`	5`-AGTACCCTTCCGCTTACCG-3`	147	61	[63]
	780433	SDHA	5`-CTACAAGGGGCAGGTTCTGA-3`	5`-AAGACAACGAGGTCCAGGAG-3`	141	61	[62]
	100628048	STX5	5`-TGCAGAGTCGTCAGAATGGA-3`	5`-CCAGGATTGTCAGCTTCTCC-3`	144	61	[21]
	110259740	TBP	5`-CTGAATGCTGAGGCGATTTC-3`	5`-GCTGTGGAGTCAGTCCTGTG-3`	186	61	[21]
	780440	YWHAZ	5`-TGATGATAAGAAAGGGATTGTGG-3`	5`-GTTCAGCAATGGCTTCATCA-3`	203	60	[15]
**GOI**	100152215	CDKN1A	5`-CATGTGGACCTGTTGCTGTC-3`	5`-TTAGGGCTTCCTCTTGGAGA-3`	168	61	[15]
	100155762	CDK1	5`-CAGCTCGCTACTCAACTCCA-3`	5`-GAGTGCCCAAAGCTCTGAAA-3`	135	61	[15]
	100154715	CDK2	5`-CGGAGCTTGTTATCGCAAAT-3`	5`-AGGGGTAGGGTTCACAAAGG-3`	143	61	[15]
	100144492	CDK4	5`-TGGTTACAAGTGGTGGGACA-3`	5`-CCACAGAAGAGAGGCTTTCG-3`	208	61	[15]

### Protein quantification

The protein content in each sample was quantified by extracting protein from a 30–100 mg aliquot of ground tissue and performing subsequent quantification by bicinchoninic acid assay (BCA). To begin, samples were homogenized in a modified protein extraction buffer adapted from “Mix2” of Alhamdani et al. 2010 [23], consisting of 20 mM HEPES, 115 mM sodium chloride, 2.4 mM potassium phosphate monobasic, and 1 mM magnesium chloride, adjusted to a pH of 7.9 and followed by subsequent addition of 1% v/v Nonidet-P40 substitute, 0.5% w/v cholic acid, and 0.25% w/v ASB-14 as detergents. A 10-fold by weight the amount of extraction buffer was added to each sample, as well as 1.0 mm zirconia/silica beads (BioSpec Products, Bartlesville, OK) to allow for tissue disruption and homogenization. The samples were then placed in a TissueLyser II (Qiagen, Hilden, Germany) and homogenized at 30 RPM for 2 min, before centrifugation at 5000 x g for 10 min to allow for the removal of the resultant supernatant. Immediately following extraction, a 25 µL aliquot of each sample was assayed in duplicate in a 96-well plate, with 200 µL of BCA reagent [24] and 10 µL of 0.1 N NaOH added to each well. Samples were diluted as needed with 0.1 N NaOH to ensure that the absorbance value and concentration within the plate fell within the 25–2000 µg/mL range of the bovine serum albumin (BSA) standard curve. After incubation for 30 min at 37 °C, the absorbance of each well at 562 nm was measured on a Spark 10M spectrophotometer (Tecan Life Sciences, Männedorf, Switzerland). Duplicate measurements for each sample were averaged, and the concentration of protein in each sample calculated using a linear equation derived from the values of the BSA standard curve.

### Fluorescent histology

A HM 325 rotary microtome was used to cut histological sections of the fetal LVR and HRT for fluorescent staining with DAPI (Thermo Fisher Scientific) and WGA, Fluorescein (Vector Laboratories, Newark, CA). Prior to staining, slides were baked and deparaffinized with xylene to remove the wax embedded in the tissue, and then subsequently rehydrated using a series of decreasing ethanol concentrations before washing in phosphate-buffered saline (PBS). Then, slides were incubated at room temperature in a 10 µg/mL solution of WGA-Fluorescein for 1 h, followed by a 10-minute room temperature incubation in 0.1 µg/mL of DAPI. After a final wash in PBS, slides were dried and cover-slipped with Mowiol 4–88 mounting media (Sigma-Aldrich, St. Louis, MO). All resultant slides were fluorescently viewed at 200X total magnification, with representative cross-sectional images taken from each sample and illumination and exposure times standardized within tissue. For the liver, two cross-sections from each fetus were cut and stained, with three images taken from each section for a total of six images per fetus. For the heart, an average of 2.58 images were taken per fetus, with the number varying based on the maximum number of representative cross-sectional areas that could be found on each slide. After imaging, analysis was done using a custom pipeline for the ImageJ software [25]. In short, WGA staining was used to manually define the largest possible artifact-free, cross-sectional area from each image, and this target region was then binarized for particle analysis. Values for threshold, particle size, and nuclei circularity were established in preliminary experiments and universally applied across all images. Results generated by the software were used to calculate nuclei density and average nuclei size as indirect measures of cellular hypertrophy and proliferation.

### Statistical analyses

Data processing and statistical analyses were carried out using the R programing language version 4.2.3 “Shortstop Beagle” [26], with visualization via the ggplot2 package [27]. Fetus was used as the experimental unit because response to *in utero* treatment is known to be impacted by fetal factors including placental efficiency and genetic potential [28]. The impact of fetal sex on all variables was initially evaluated and found to have no significant effect, and was thus removed from subsequent analyses. Gene expression data was largely heteroskedastic and/or not normally distributed and was thus assessed using nonparametric Wilcoxon signed-ranked test of the ΔCT values calculated as the difference between the cycle threshold for each gene of interest and the geometric mean of two to three stable reference genes within tissue. The impact of hypothyroidism on protein content across tissue was evaluated using a linear mixed effect model from the package nlme [29], including tissue and treatment as fixed effects and dam as a random effect. The estimated marginal mean of the CON group in each tissue was used to compare protein content between tissues using the package emmeans [30] with a Bonferroni *P*-value correction. Nuclear size and density were assessed using a standard linear model. The threshold for significance was *p* < 0.05, with values between 0.05 and 0.1 reported as trends.

## Results

### Gene expression

To evaluate the effect of hypothyroidism on late-gestation development of key fetal organs, the expression of three cell cycle promoters (CDK1, CDK2, and CDK4) and one cell cycle inhibitor (CDKN1A) was measured across eight fetal tissues. The stable reference genes used for each tissue were ACTB, STX5, and GAPDH in the HRT, ACTB, YWHAZ, and GAPDH in the ILE, ACTB, YWHAZ, and STX5 in the KID, YWHAZ and TBP in the LNG, YWHAZ, RPL19, and HMBS in the LVR, YWHAZ and GAPDH in the MUS, YWHAZ and STX5 in the SPLN, and ACTB and STX5 in the THY.

**Fig. 1:**
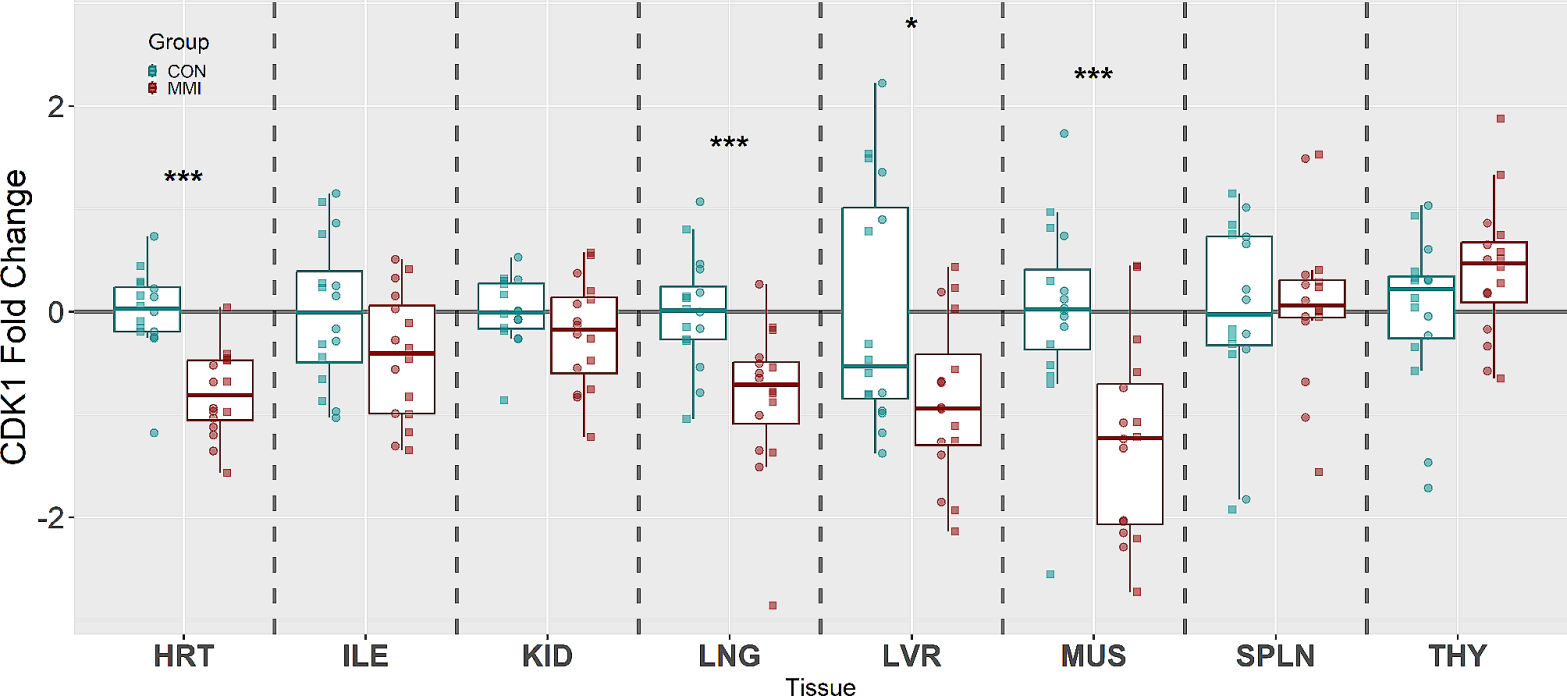
Expression of CDK1 in heart (HRT), ileum (ILE), kidney (KID), lung (LNG), liver (LVR), loin muscle (MUS), spleen (SPLN), and thymus (THY) tissue derived from fetuses at day 106 of gestation following 21 days of control (CON, *n* = 16) or methimazole (MMI, *n* = 16) treatment. Fold changes were calculated within tissue relative to the average of the CON group and presented on a log10 axis to visualize both up and down regulations. *P*-values were calculated using a Wilcoxon signed-rank test with statistical differences denoted as *p < 0.05, ** *p* < 0.01, and ***p < 0.001 within a given tissue. Data is presented in standard boxplots displaying the median and standard quartiles with individual data points overlayed identifying male and female fetuses with □ and ○, respectively

CDK1, which is responsible for the transition from the G2 to M phase of the cell cycle, was significantly downregulated in four of the eight tissues studied when comparing the MMI group to the CON (Fig. 1). The largest decrease in fold change was observed in MUS (x̃ = -1.53 fold, *P* < 0.001), followed sequentially by LVR (x̃ = -1.38 fold, *P* = 0.043), HRT (x̃ = -1.32 fold, *P* < 0.001), and LNG (x̃ = -1.28 fold, *P* < 0.001). The other four tissues, ILE, KID, SPLN, and THY, showed no significant changes between experimental groups (*P* = 0.119, 0.270, 0.780, and 0.184, respectively).

**Fig. 2:**
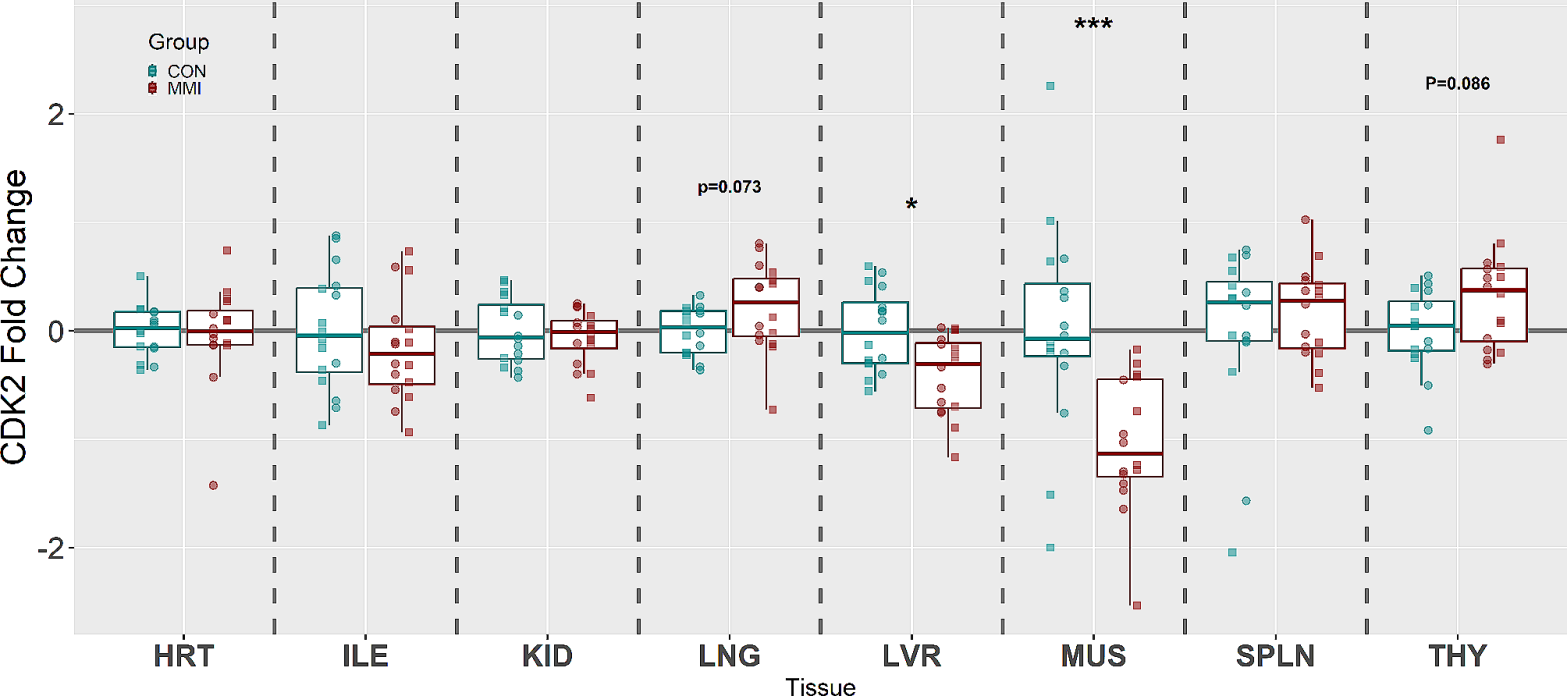
Expression of CDK2 in heart (HRT), ileum (ILE), kidney (KID), lung (LNG), liver (LVR), loin muscle (MUS), spleen (SPLN), and thymus (THY) tissue derived from fetuses at day 106 of gestation following 21 days of control (CON, *n* = 16) or methimazole (MMI, *n* = 16) treatment. Fold changes were calculated within tissue relative to the average of the CON group and presented on a log10 axis to visualize both up and down regulations. *P*-values were calculated using a Wilcoxon signed-rank test with statistical differences denoted as *p < 0.05, ** *p* < 0.01, and ***p < 0.001 within a given tissue. Data is presented in standard boxplots displaying the median and standard quartiles with individual data points overlayed identifying male and female fetuses with □ and ○, respectively

CDK2, which promotes the transition from the G1 to S phase of the cell cycle, was significantly downregulated in both the MUS (x̃ = -1.48 fold, *P* < 0.001) and LVR (x̃ = -1.11 fold, *P* = 0.014) tissues of MMI-treated fetuses (Fig. 2), which is similar to the downregulations of CDK1 seen in these tissues. In addition, the LNG (x̃ = 1.10 fold, *P* = 0.073) and THY (x̃ = 1.14 fold, *P* = 0.086) tissues of the MMI-treated fetuses showed a trend towards upregulation of this gene relative to CON, while the HRT, ILE, KID, and SPLN showed no significant changes (*P* = 0.752, 0.381, 0.669, and 0.985, respectively).

**Fig. 3:**
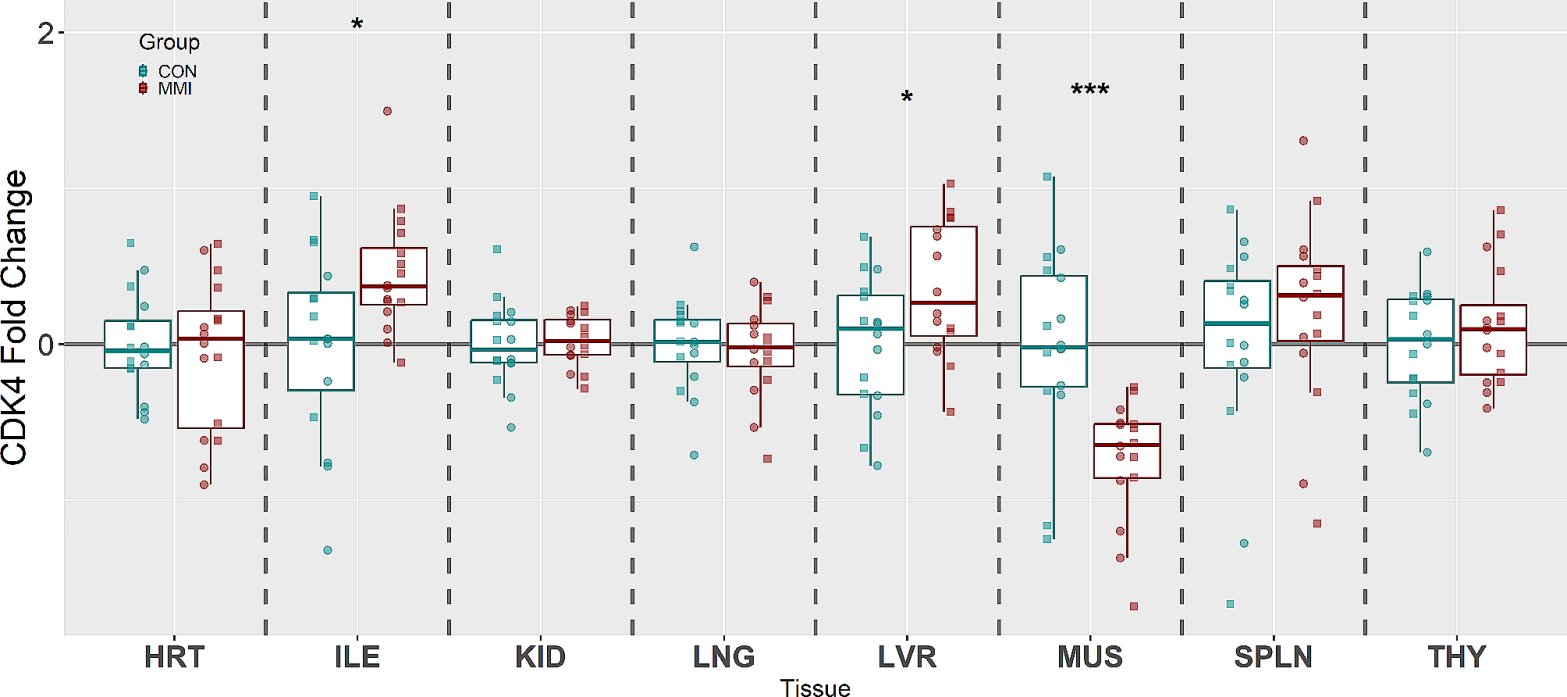
Expression of CDK4 in heart (HRT), ileum (ILE), kidney (KID), lung (LNG), liver (LVR), loin muscle (MUS), spleen (SPLN), and thymus (THY) tissue derived from fetuses at day 106 of gestation following 21 days of control (CON, *n* = 16) or methimazole (MMI, *n* = 16) treatment. Fold changes were calculated within tissue relative to the average of the CON group and presented on a log10 axis to visualize both up and down regulations. *P*-values were calculated using a Wilcoxon signed-rank test with statistical differences denoted as *p < 0.05, ** *p* < 0.01, and ***p < 0.001 within a given tissue. Data is presented in boxplots displaying the median and standard quartiles with individual data points overlayed identifying male and female fetuses with □ and ○, respectively

CDK4, which pushes the cell cycle out of G0 into the G1 or S phase, was significantly upregulated in the ILE (x̃ = 1.14 fold, *P* = 0.035) and LVR (x̃ = 1.10 fold, *P* = 0.039) tissues of MMI-treated fetuses relative to CON, while being downregulated in MUS tissue (x̃ = -1.25 fold, *P* < 0.001) (Fig. 3). The other five tissues, HRT, KID, LNG, SPLN, and THY, showed no significant changes in expression of CDK4 (*P* = 0.956, 0.539, 0.696, 0.361, and 0.616, respectively).

**Fig. 4:**
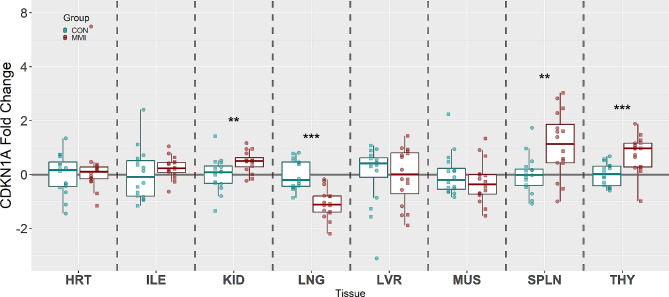
Expression of CDKN1A in heart (HRT), ileum (ILE), kidney (KID), lung (LNG), liver (LVR), loin muscle (MUS), spleen (SPLN), and thymus (THY) tissue derived from fetuses at day 106 of gestation following 21 days of control (CON, *n* = 16) or methimazole (MMI, *n* = 16) treatment. Fold changes were calculated within tissue relative to the average of the CON group and presented on a log10 axis to visualize both up and down regulations. *P*-values were calculated using a Wilcoxon-signed rank test with statistical differences denoted as *p < 0.05, ** *p* < 0.01, and ***p < 0.001 within a given tissue. Data is presented in boxplots displaying the median and standard quartiles with individual data points overlayed identifying male and female fetuses with □ and ○, respectively

Lastly, the expression of CDKN1A, a potent cell cycle inhibitor, showed significant changes in four of the eight tissues analyzed (Fig. 4). CDKN1A was significantly upregulated in the KID (x̃ = 1.19 fold, *P* = 0.006), SPLN (x̃ = 1.49 fold, *P* = 0.005), and THY (x̃ = 1.40 fold, *P* < 0.001) tissues of MMI-treated fetuses relative to CON, while being uniquely downregulated in the LNG of MMI-treated fetuses (x̃ = -1.47 fold, *P* < 0.001). The other 4 tissues, HRT, ILE, LVR, and MUS, showed no significant changes in expression (*P* = 0.985, 0.254, 0.926, and 0.305, respectively).

### Protein quantification

**Fig. 5:**
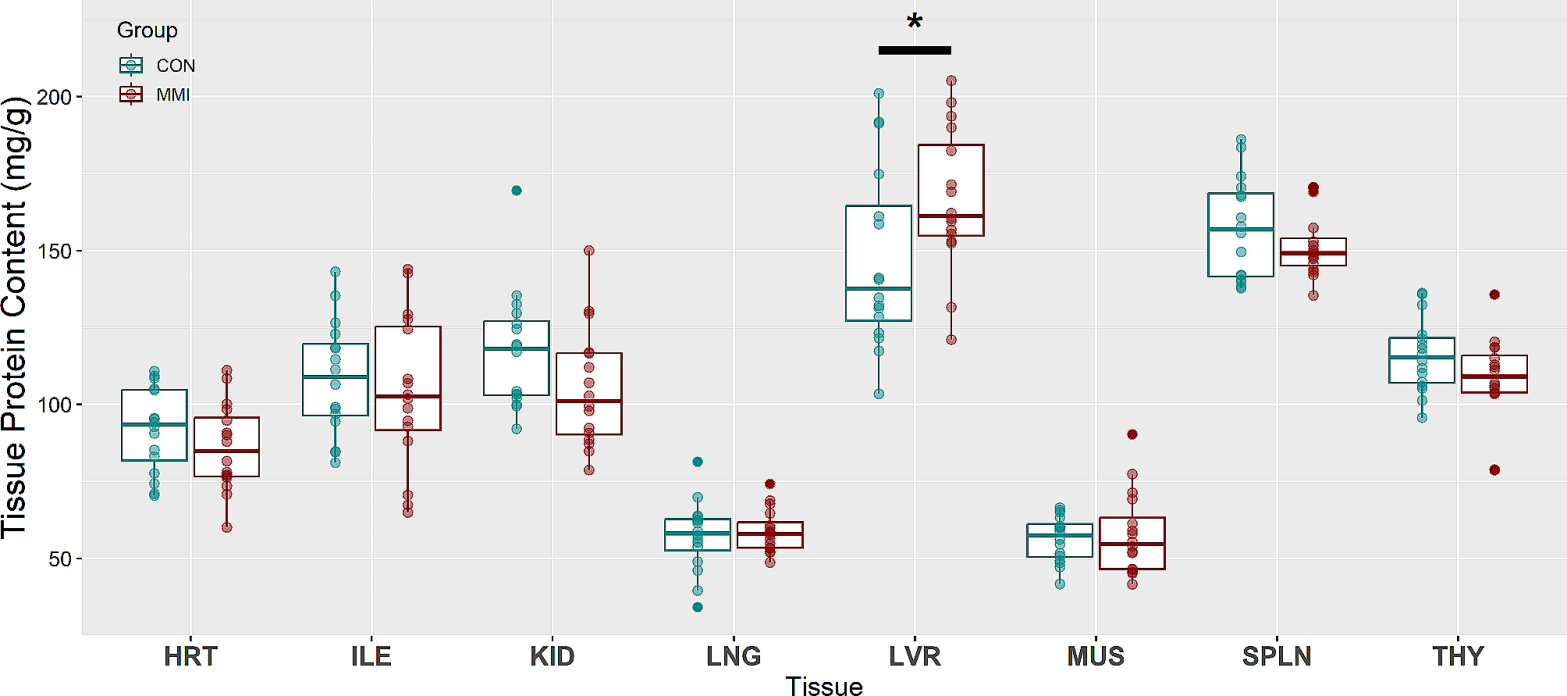
Protein content of heart (HRT), ileum (ILE), kidney (KID), lung (LNG), liver (LVR), loin muscle (MUS), spleen (SPLN), and thymus (THY) tissue derived from fetuses at day 106 of gestation following 21 days of control (CON, *n* = 16) or methimazole (MMI, *n* = 16) treatment. Data were evaluated using a linear mixed effect model with tissue and treatment as fixed effects and dam as a random effect, with * denoting a statistical difference of *p* < 0.05. Data is presented in boxplots displaying the median and standard quartiles with individual data points overlayed

The protein content of each fetal tissue sample was quantified using BCA assay to allow for comparison of protein concentration across tissues, as well as to evaluate the impact of late-gestation hypothyroidism on tissue protein concentration. Of the eight tissues analyzed, only the LVR exhibited a significant change in protein concentration as a result of treatment, with the mean of the MMI-treated fetuses being 19.34 milligrams greater than the mean for fetuses in the CON group (*P* = 0.036, Fig. 5).

Under the method used in this study, and specifically focusing on the CON groups, the SPLN averaged 157.1 mg/g of protein, which was significantly higher than the protein content of the HRT, ILE, KID, LNG, MUS, and THY tissues (*P* < 0.0001 for all tissues), but not significantly higher than the protein content of the LVR (*P* = 1.000). The LVR averaged 147.1 mg/g of protein, which, similar to the SPLN, was significantly higher than that of the HRT, ILE, KID, LNG, MUS, and THY tissues (*P* < 0.0001 for all tissues). The KID and THY averaged 117.4 and 115.9 mg/g of protein, respectively, the difference of which was nonsignificant (*P* = 1.000). Consequently, the protein content of both KID and THY was significantly greater than that of the HRT (KID – HRT: *P* = 0.0003; THY – HRT: *P* = 0.0009), LNG (*P* < 0.0001 for both tissues), and MUS (*P* < 0.0001 for both tissues), but not the ILE (*P* = 1.000 for both tissues). The ILE averaged 108.5 mg/g of protein, which was only significantly greater than the LNG and MUS tissues (*P* < 0.0001), and not the HRT (*P* = 0.102). The last two tissues, LNG and MUS, averaged 57.2 and 56.2 mg/g of protein, respectively. As stated previously, these values were significantly lower than the averages for all other tissues, but the difference between LNG and MUS was found to be nonsignificant (*P* = 1.000).

### Histology

**Fig. 6:**
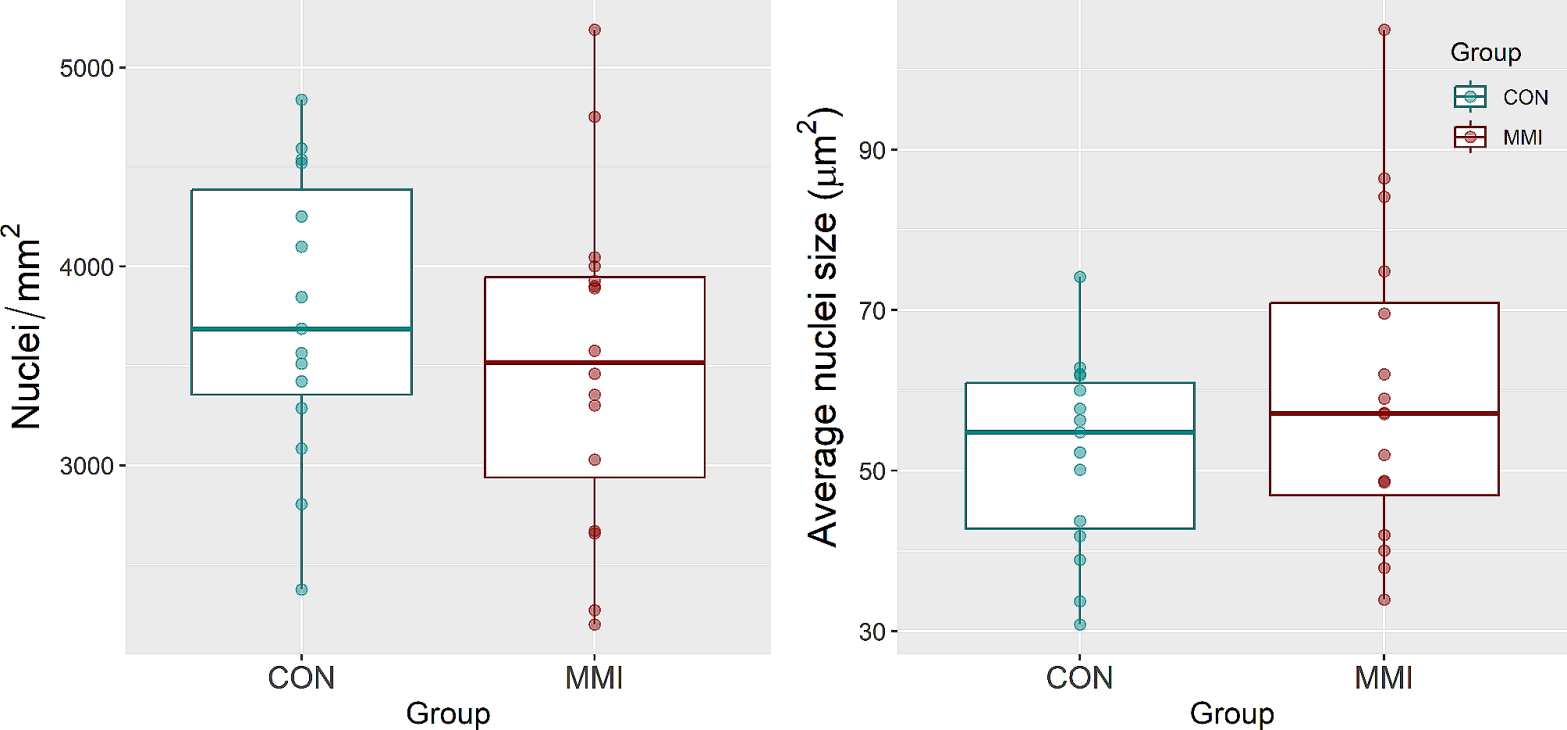
Nuclei density and average nuclei size of heart tissue derived from fetuses at day 106 of gestation following 21 days of control (CON, *n* = 16) or methimazole (MMI, *n* = 16) treatment. Data were evaluated using a standard linear model, with no significant differences (*p* < 0.05) detected for either measure. Data is presented in boxplots displaying the median and standard quartiles with individual data points overlayed

**Fig. 7:**
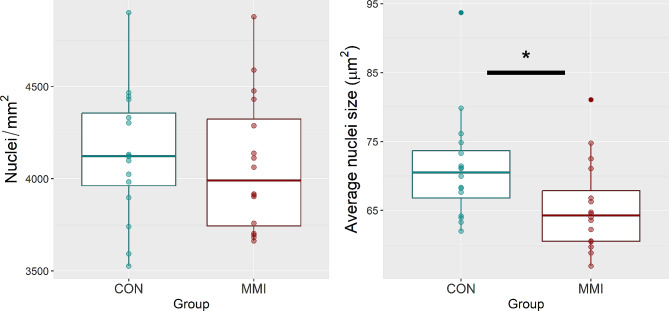
Nuclei density and average nuclei size of liver tissue derived from fetuses at day 106 of gestation following 21 days of control (CON, *n* = 16) or methimazole (MMI, *n* = 16) treatment. Data were evaluated using a standard linear model, with * denoting a statistical difference of *p* < 0.05. Data is presented in boxplots displaying the median and standard quartiles with individual data points overlayed

To indirectly examine the HRT and LVR for evidence of changes in cellular hypertrophy and/or proliferation, average nuclei density and nuclei size was quantified for each tissue. Histological analysis of fluorescently stained cardiac cross-sections showed no significant changes in average nuclei density (*P* = 0.383) or nuclei size (*P* = 0.260) between the MMI and CON groups (Fig. 6). In the LVR, a significant decrease in average nuclei size of the MMI group relative to CON was observed (*P* = 0.025, Fig. 7), but similar to in the HRT, there was no change in average nuclei density seen in the LVR (*P* = 0.659).

## Discussion

Thyroid hormones are a critical part of the maternal-fetal milieu, regulating metabolism and serving a fundamental role in promoting fetal growth and the development of vital organ systems. A healthy human pregnancy is associated with a substantial upregulation in thyroid activity [31]; however, thyroid function is among the most frequently disrupted endocrine pathways during human pregnancy [3]. A similar susceptibility to such perturbations has been observed in pregnant swine [15] and horses [32]. Such disruptions have been shown to have widespread effects on the development and physiology of organ systems including the cardiovascular [33], renal [34], pulmonary [35], hepatic [34], musculoskeletal [36], and other endocrine [37] systems. Therefore, rather than targeting an individual system, the present study sought to understand the more global effects of fetal hypothyroidism on cell division using a non-pathogenic porcine model. In contrast to many rodent studies of fetal hypothyroidism [38, 39], the model presented here is unique in that hypothyroidism was only observed in the MMI-treated fetuses, with no significant effects of treatment observed in the dams [14].

To accomplish this analysis with the desired breadth and depth, we initially utilized targeted gene expression analysis to evaluate cell cycle regulation across eight fetal organs from 32 fetuses for a total of 256 unique samples. Assessment of cell division within tissues is most commonly conducted via immunohistochemical staining for Ki67 [40] or proliferating cell nuclear antigen (PCNA) [41]. These staining methods, however, were unfeasible given the large sample size used in this study, which necessitated the use of cell-cycle analysis by qPCR instead. The chosen gene targets CDK1, CDK2, and CDK4 are cell cycle promoters which are each known to act in a particular phase of the cell cycle, but may be regulated through non-transcriptional mechanisms including cyclin subunit binding, phosphorylation, or inhibitory subunit activity [42]. The complexity of such regulation is a potential caveat to analysis of these genes by qPCR. CDKN1A, commonly referred to as P21, however, is one of the most well-understood genes and is known to be regulated at the transcriptional level [43], making it a viable target for qPCR analysis. Additionally, the effects of CDKN1A on inhibiting cell cycle progression and being closely linked with the apoptotic pathway are well defined in established literature [44].

In addition to analysis by qPCR, the HRT and LVR were histologically examined for evidence of changes in cellular proliferation and/or hypertrophy by measuring average nuclei size and density. As cells prepare for mitosis, they must replicate and decondense their DNA, and it has been suggested that a larger nuclear size in G1 is highly and positively correlated with the subsequent rate of DNA synthesis in the S stage, with nuclear size also being a potential factor in the G1-S cell cycle transition [45]. This suggests that cells with smaller nuclei are not synthesizing the required DNA for subsequent mitotic division and could thus be considered to be proliferating at a reduced rate. This concept has been corroborated in conjunction with Ki67 staining, which showed that tumor cells with Ki67-negative nuclei have a smaller nuclear area compared to cells with Ki67-positive nuclei [46], indicating that nuclear area is a key indicator of proliferation status. In addition, nuclei density may be used as a measure of cellular hypertrophy, as cells that undergo hypertrophy and are larger in size should have individual nuclei that are farther apart and thus less dense [47].

Following the above logic, the LVR showed no signs of changes in hypertrophy as a result of treatment, but the decrease in average nuclei size in the LVR as a result of MMI treatment is a potential indicator of reduced rates of cellular proliferation, indicating that hypoplasia of the LVR may be occurring. This is supported by the coincidental downregulations seen in hepatic CDK1 and CDK2, but not by the upregulation seen in CDK4. Additionally, the LVR tissue of MMI-treated fetuses contained significantly more protein than that of the CON. During this late stage of gestation, the crude protein percentage of the LVR is relatively stable, but trends downward in healthy euthyroid fetuses [20]. Thus, one possible explanation of the increase in LVR protein content seen in the MMI-treated fetuses is delayed organ development. The method by which protein was analyzed, however, differs between our study and the previously mentioned one, as we quantified protein via BCA assay, while the other study quantified protein on a dry matter basis using the combustion method. Thus, there is significant variation in the reported protein percentage values, which could be explained by the fact that the crude protein percentage of the other study was calculated as a percentage of dry matter, while our method of quantifying protein utilized flash-frozen tissue samples that were not freeze-dried prior to quantification. As water content across tissues may vary, and the possibility of alterations in tissue water content as a result of fetal hypothyroidism cannot be discounted, future research is needed to verify the results of the current measurements on a dry matter basis.

PRRSV is among the most significant pathogens impacting global pork production. The responsible etiological agent is a single-stranded RNA virus capable of crossing the late gestation placenta and resulting in a combination of fetal death and the birth of weak, underdeveloped offspring. Of pertinence, fetal infection with PRRSV is known to cause significant decreases in circulating T3 and T4 [15], caused at least in part by altered deiodinase activity within the fetal KID and LVR, along with the maternal fetal interface [48]. This combination of viral infection and hypothyroidism is coincident with the suppression of cell division across multiple organs [16], but despite an identical gestational age and length of disruption between studies, the organs most severely impacted by PRRSV-induced hypothyroidism were the HRT and KID [16]. This apparent incongruity with our data suggests that cell division in the HRT and KID is more readily impacted by viral infection than thyroid status. The relatively scarce impact of goitrogen-induced fetal hypothyroidism in the HRT is also evidenced by the results of the histological study presented here, in which the HRT tissue of MMI-treated fetuses showed no evidence of changes in cellular hyperplasia or hypertrophy/hypotrophy in comparison to the CON group.

The success of the swine industry is dependent on efficient postnatal production of skeletal muscle, and as hyperplasia within muscle cells is temporally restricted to the *in utero* period, proper fetal development is critical from an industry perspective. Various factors such as uterine crowding and subsequent intrauterine growth retardation [49], maternal nutrition [50], and the maternal environment [51] have all been previously shown to affect muscle development *in utero*. Here, we saw distinct disruptions in MUS cell cycle regulation as a result of MMI-induced hypothyroidism, with significantly decreased expression of all the studied cell cycle promoters (CDK1, 2, and 4) in the hypothyroid fetuses, suggesting that congenital hypothyroidism may also negatively impact prenatal muscle development. This apparent cell cycle suppression without a corresponding increase in CDKN1A is consistent with prior findings regarding PRRSV-induced fetal hypothyroidism [16], which suggests that PRRSV may indirectly supress muscle cell proliferation *in utero* by first reducing circulating thyroid hormone levels. However, the timing of muscle cell hyperplasia *in utero* was previously reported to conclude between days 85–95 of gestation [52], indicating that the cell cycle should no longer be altered at the late-gestational time point used in this study. Thus, a possible explanation for the observed transcriptional changes is that the changes in gene expression are a result of changes in a population of satellite cells [53], not a result of changes in myofibers, or that muscle cell hyperplasia occurs later in gestation than originally reported.

The SPLN and THY both act as nurseries for vital immune cells, making their successful development paramount to health and survival following postnatal exposure to pathogens. In the developing fetus, immature T cells, known as thymocytes, migrate to the THY to undergo maturation into functional T cells [54], while the SPLN aids in the maturation and proliferation of B cells [55]. Previous research in rats [56] and fish [57] has shown that treatment with antithyroid drugs modulates immune function in developing animals, suggesting that thyroid hormones are essential for SPLN and thymic fetal development. In our study, we observed an increase in CDKN1A expression in the SPLN and THY of MMI-treated fetuses, which could be indicative of reduced proliferation that has the potential to weaken the postnatal adaptive immune response, further corroborating the important role of thyroid hormones in immune system development.

Finally, in the late-gestational timepoint studied here, successful terminal maturation of various fetal organs such as the LNG, LVR, KID, and gastrointestinal tract involves the endogenous release of glucocorticoids, mainly, cortisol [58]. In the LNG, cortisol release results in the initiation of pulmonary surfactant production, as well as an overall reduction in cell division [59], both of which must occur to allow for a viable transition to extrauterine life. As a result of MMI-treatment, fetuses in the present study experienced a significant decrease in both LNG CDK1 expression and CDKN1A expression, the latter of which was unique to this tissue. While the decrease in CDK1 could signify reduced proliferation, the apparent decrease in CDKN1A is instead evident of a decrease in cell cycle suppression and possible increased proliferation, which is the opposite of what should be occurring in the LNG during this time. This period of terminal maturation, however, generally results in increased activity of the fetal gastrointestinal tract, as cortisol secretion stimulates factors such as brush-border enzyme production [60], and the small intestine continually increases in both circumference and weight [61], which would suggest possible changes in cellular proliferation. Interestingly, the effects of MMI-treatment in the current study appeared to be least severe in the gastrointestinal tract, with only a slight upregulation of CDK4 seen in the ILE tissue of MMI-treated fetuses.

## Conclusions

Collectively, our results show evidence of widespread alterations in the expression of key cell cycle regulator genes as a result of MMI-induced congenital hypothyroidism, with LVR and MUS experiencing the most changes in gene expression. These observed alterations in cell cycle progression are evidence of detrimental effects of hypothyroidism on the developing fetus at a molecular level, the longer-term implications of which may include a weakened postnatal adaptive immune response, inhibited terminal maturation of the LNG, and, of vital importance to the swine industry, a negative impact on MUS development. Future research is required to better understand the meaning of the increase in LVR protein content resulting from MMI-treatment, as well as to better understand the more long-term implications of these cell cycle disruptions into and beyond the postnatal period.

## Data Availability

The datasets generated during and/or analyzed during the current study are available from the corresponding author on reasonable request.

## Abbreviations

T3: Triiodothyronine
T4: Thyroxine
G0: Gap 0 phase of the cell cycle
G1: Gap 1 phase of the cell cycle
S: DNA synthesis stage of the cell cycle
G2: Gap 2 phase of the cell cycle
M: Mitosis phase of the cell cycle
HRT: Fetal heart
ILE: Fetal ileum
KID: Fetal kidney
LNG: Fetal lung
LVR: Fetal liver
MUS: Fetal longissimus dorsi muscle
SPLN: Fetal spleen
THY: Fetal thymus
CON: Euthyroid fetuses from dams given daily sham carrier treatment during days 85–106 of gestation
MMI: Hypothyroid fetuses from dams given daily methimazole treatment during days 85–106 of gestation
CDK1: Cyclin dependent kinase 1
CDK2: Cyclin dependent kinase 2
CDK4: Cyclin dependent kinase 4
CDKN1A: Cyclin dependent kinase inhibitor 1A
PRRSV: Porcine reproductive and respiratory syndrome virus
